# Precipitation overrides warming in mediating soil nitrogen pools in an alpine grassland ecosystem on the Tibetan Plateau

**DOI:** 10.1038/srep31438

**Published:** 2016-08-16

**Authors:** Li Lin, Biao Zhu, Chengrong Chen, Zhenhua Zhang, Qi-Bing Wang, Jin-Sheng He

**Affiliations:** 1 Department of Ecology, College of Urban and Environmental Sciences, and Key Laboratory for Earth Surface Processes of the Ministry of Education, Peking University, 100871, Beijing, China; 2 Environmental Futures Research Institute, Griffith School of Environment, Griffith University, 4111, Nathan, Queensland, Australia; 3 Key Laboratory of Adaptation and Evolution of Plateau Biota, Northwest Institute of Plateau Biology, Chinese Academy of Sciences, 23 Xinning Rd., 810008 Xining, China; 4 State Key Laboratory of Vegetation and Environmental Change, Institute of Botany, Chinese Academy of Sciences, 100093, Beijing, China

## Abstract

Soils in the alpine grassland store a large amount of nitrogen (N) due to slow decomposition. However, the decomposition could be affected by climate change, which has profound impacts on soil N cycling. We investigated the changes of soil total N and five labile N stocks in the topsoil, the subsoil and the entire soil profile in response to three years of experimental warming and altered precipitation in a Tibetan alpine grassland. We found that warming significantly increased soil nitrate N stock and decreased microbial biomass N (MBN) stock. Increased precipitation reduced nitrate N, dissolved organic N and amino acid N stocks, but increased MBN stock in the topsoil. No change in soil total N was detected under warming and altered precipitation regimes. Redundancy analysis further revealed that soil moisture (26.3%) overrode soil temperature (10.4%) in explaining the variations of soil N stocks across the treatments. Our results suggest that precipitation exerted stronger influence than warming on soil N pools in this mesic and high-elevation grassland ecosystem. This indicates that the projected rise in future precipitation may lead to a significant loss of dissolved soil N pools by stimulating the biogeochemical processes in this alpine grassland.

Nitrogen (N) plays an important role in mediating ecosystem functions in many ecosystems^1^,^2^,^3^. Even small changes in soil N pools could have severe influences on both carbon (C) and N cycling^4^,^5^. Soils in Tibetan alpine grasslands, which store large amounts of N^6^,^7^,^8^,^9^, have experienced a dramatic rise in temperature and a change in precipitation in the past several decades^10^,^11^,^12^. However, the changes of soil N pools in the Tibetan alpine grasslands under different climate change scenarios remain elusive.

Warming and altered precipitation regimes are two main components of climate change, which have biotic or abiotic paths to affect soil N pools in the grassland ecosystems. On the one hand, climate change could shift plant mortality to mediate the soil N input^4^,^13^ and also impact microbial biomass and activities to control N_2_O emission rate considered as soil N output^3^. On the other hand, changes in soil properties, such as particle size, pH and water holding capability, may also affect conversions of different forms of nitrogen under climate change regimes^14^,^15^. A number of studies have examined the effects of experimental warming on soil N pools in China’s grasslands^13^,^16^,^17^,^18^,^19^,^20^,^21^,^22^,^23^ and in the arctic regions^24^,^25^. In general, weak effects of warming on the concentrations of soil total N (STN) and some labile N pools have been observed in the warming experiments in the Inner Mongolian^16^,^22^,^23^ and the Tibetan grasslands^19^. What’s more, a global meta-analysis on terrestrial N pools showed that the response of soil total N stock in grasslands to experimental warming was little^26^. As for the labile N pools, the results vary with the components of soil N studied. In the alpine, subalpine, or arctic ecosystems, the effects of warming may have different directions on soil labile N pools^27^. First, warming could promote microbial enzyme activity and soil particle fragmentation, and therefore enhance soil labile N stocks^21^,^26^. For example, warming increased soil NH_4_^+^-N concentration in two grassland experiments in the East of Tibetan Plateau^21^. In the global grassland/prairie ecosystems, warming showed a significantly positive effect on inorganic N pools^26^. Secondly, warming could accelerate N_2_O emission by stimulating microbial denitrification and gas transportation, which may decrease soil NO_3_^−^-N concentration in soils^20^.

A few studies on how altered precipitation affects soil N pools have been conducted in the grassland ecosystems, which showed that precipitation is a key factor in shaping the pattern of soil N pools^28^. For example, water addition significantly increased soil NO_3_^−^-N and soil inorganic N (SIN) in a semi-arid temperate steppe of northern China^16^,^29^,^30^. This could result from enhanced microbial activities in response to higher water availability^28^. By contrast, water addition decreased soil N pools indirectly by enhancing net primary production (NPP) in a tall grass prairie^31^. Nevertheless, Jongen *et al*.^32^ reported that soil N pools were not significantly affected by precipitation variability in an oak woodland. Therefore, we need further studies on the effects of altered precipitation, particularly the interactions between warming and altered precipitation on soil N pools in the Tibetan alpine grassland.

Previous studies have mostly focused on STN and SIN, but ignored other forms of soil labile N, such as soil dissolved organic N (DON). However, DON, especially amino acid N (AAN), is an important source of N for plants in the alpine or arctic ecosystems^33^,^34^,^35^,^36^. Dominant plant species, such as *Kobresia humilis, Saussurea superba* and *Stipa aliena*, in the Tibetan alpine grassland have been reported to acquire organic N which contributed up to 30% of the total N uptake^37^. Thus, the responses of DON to climate change should be considered particularly in the alpine ecosystems on the Tibetan Plateau, which have a large soil N stock with plants being able to obtain DON directly.

To better understand how warming, altered precipitation and their interactions affect soil N pools in an alpine grassland, we conducted a field experiment to investigate the changes in six soil N stocks in the topsoil (0–20 cm), the subsoil (40–70 cm) and the entire soil profile (0–70 cm). Considering the previous results that found temperature as the factor controlling nutrients cycling and functions of the alpine ecosystem^12^,^13^,^27^, we hypothesized that (1) soil labile N pools responded to warming and its interaction with altered precipitation in the short period, and (2) temperature is the main driver to the changes. Our results will lead us to generate new insights into effects of climate change on soil N pools in alpine grassland ecosystems and to predict trends of soil N pools in the future.

## Results

### Soil temperature and moisture

**Figure 1:**
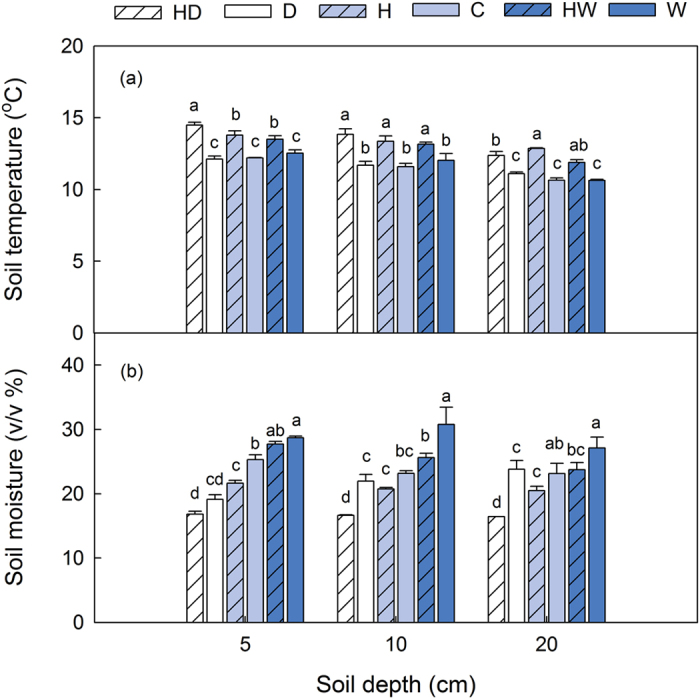
Soil temperature (°C) **(a)** and soil moisture (v/v %) **(b)** at depths of 5 cm, 10 cm and 20 cm under warming and altered precipitation regimes during the growing season of 2013. C, control; H, heated; D, dry; HD, heated and dry; W, wet; HW, heated and wet. Different letters mean significant differences between treatments at *P* < 0.05 level for each soil depth. Mean ± SE is shown in the figure.

During the growing season in 2013, soil temperature and moisture at 5, 10 and 20 cm depths across treatments were shown in Fig. 1. Warming significantly increased soil temperature at all three depths (Fig. 1a). Compared with the unheated plots, increases in seasonal mean soil temperature at 5, 10 and 20 cm depths in the heated plots were 1.64, 1.68 and 1.58 °C, respectively. In contrast, the main effects of altered precipitation and the interaction between warming and altered precipitation on soil temperature were not statistically significant.

We observed significant effects of warming, altered precipitation and their interaction on soil moisture (Fig. 1b). Warming showed significantly negative effects on soil moisture. Both warming and dry treatments reduced soil moisture. As expected, wet treatment increased soil moisture by 3.6–6.2%. In addition, mean soil moisture in the wet plots were up to 27.1–30.8% at 5 cm depth approaching water holding capacity in this mesic grassland.

### Soil N pools

Table 1 summarized the LMM (Linear Mixed Model) results on the main and interactive effects of warming and altered precipitation on soil N stocks. We did not find significant interactions between warming and altered precipitation on all soil N stocks at each depth (Table 1; Table S1).

**Figure 2:**
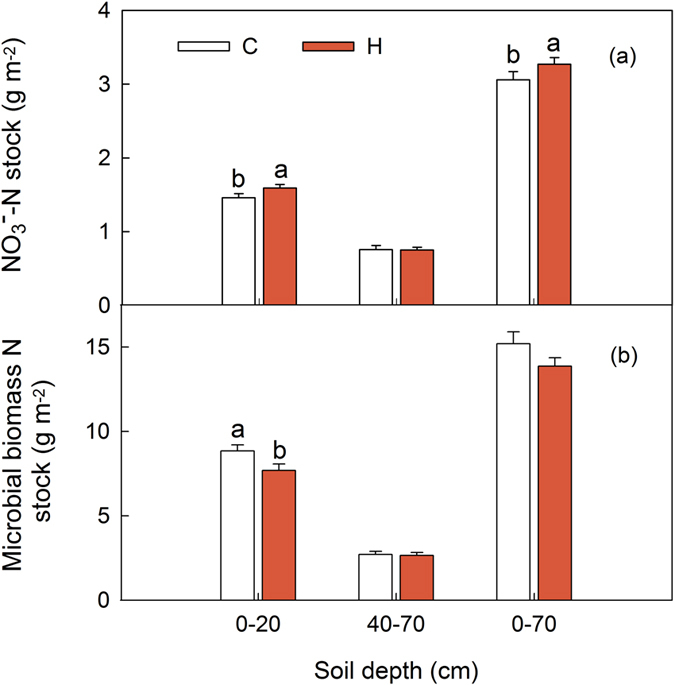
Effects of warming on NO_3_^−^-N **(a)** and microbial biomass **N (b)** stocks in the topsoil, the subsoil and the entire soil profile. C, control; H, heated. Different letters mean significant differences between factors at *P* < 0.05 for each soil depth. Mean ± SE is shown in the figure.

Warming did not affect the stocks of STN, NH_4_^+^-N, DON and AAN at each depth, but had significant effects on NO_3_^−^-N and MBN stocks (Fig. 2). In the topsoil, NO_3_^−^-N stock in the heated plots was 8.9% higher than that in the unheated plots, and MBN stock in the heated plots was 13.0% lower than that in the unheated plots. In the entire soil profile, warming significantly elevated NO_3_^−^-N stock by 6.9%, compared with the unheated plots.

**Figure 3:**
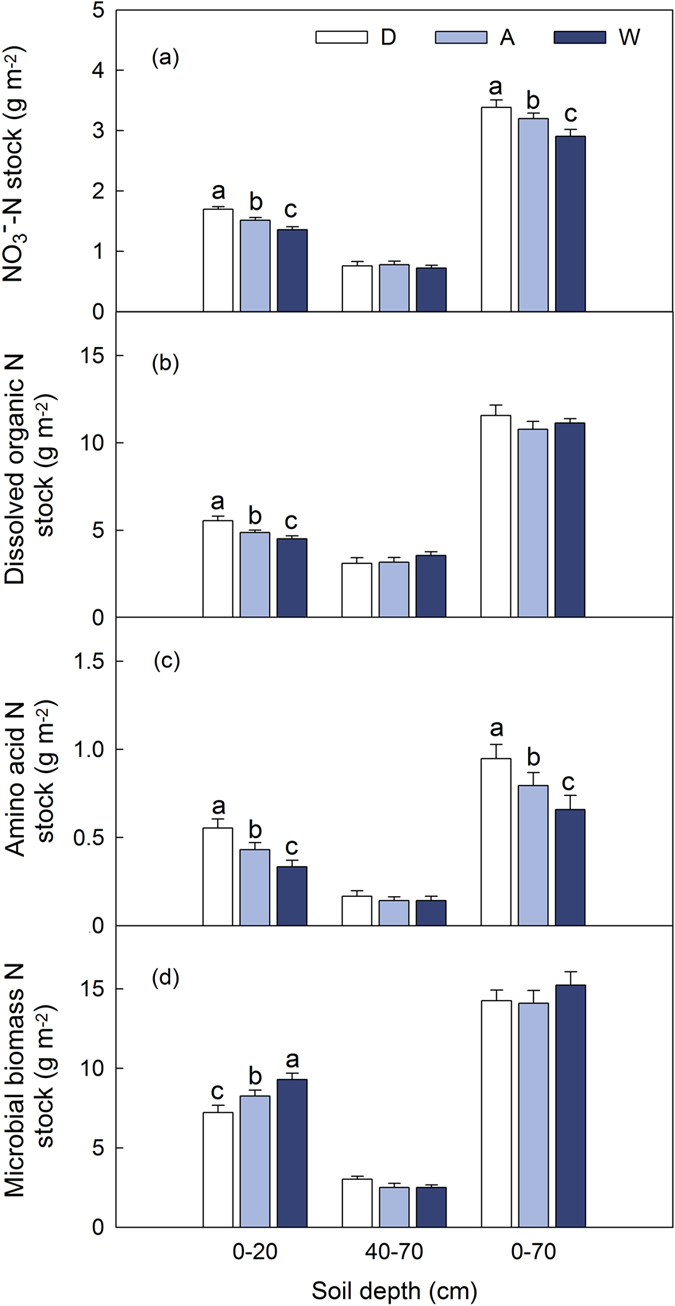
Effects of altered precipitation on NO_3_^−^-N **(a)**, dissolved organic N (2 M KCl extracts) **(b)**, amino acid N **(c)** and microbial biomass N **(d)** stocks in the topsoil, the subsoil and the entire soil profile. A, ambient; D, dry; W, wet. Different letters mean significant differences between factors at *P* < 0.05 for each soil depth. Mean ± SE is shown in the figure.

Altered precipitation had no effects on the stocks of STN and NH_4_^+^-N, but significantly affected dissolved N (i.e. NO_3_^−^-N, DON and AAN) stocks in the topsoil (Fig. 3). In the topsoil, we observed decreases in all dissolved N stocks and an increase in MBN stock in the wet plots. In the entire soil profile, both NO_3_^−^-N and AAN stocks were significantly changed among precipitation treatments.

### The pattern of soil N pools and its drivers

**Figure 4:**
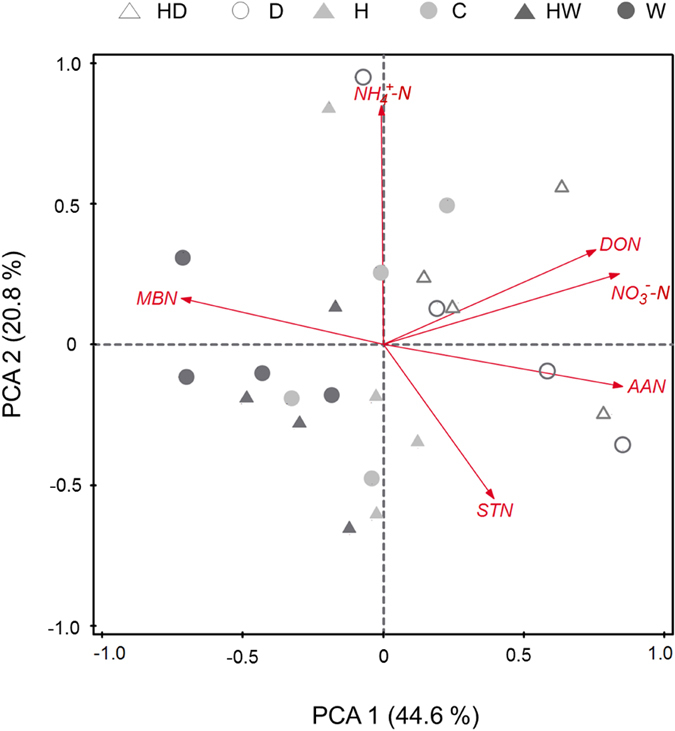
Biplot of principal components analysis (PCA) of six soil N stocks in the topsoil under warming and altered precipitation regimes. C, control; H, heated; D, dry; HD, heated and dry; W, wet; HW, heated and wet. STN, soil total nitrogen; NH_4_^+^-N, ammonium-N; NO_3_^−^-N, nitrate-N; DON, dissolved organic N (2 M KCl extracts); AAN, amino acid N; MBN, microbial biomass N.

Principal components analysis (PCA) was used to analyze the pattern of soil N pools in the topsoil (Fig. 4; Table S2). Axes 1 and 2 explained 44.6% and 20.8% of the total variation, respectively. In the ordination plot, sample points from the heated plots overlapped those from the unheated plots, while sample points from the dry or wet plots were separated along the Axis 1. In addition, arrows of dissolved N pools had the same directions, which were opposite to the arrow of MBN, indicating that impacts of warming and altered precipitation on dissolved N pools may be different from the effects on MBN pool.

**Figure 5:**
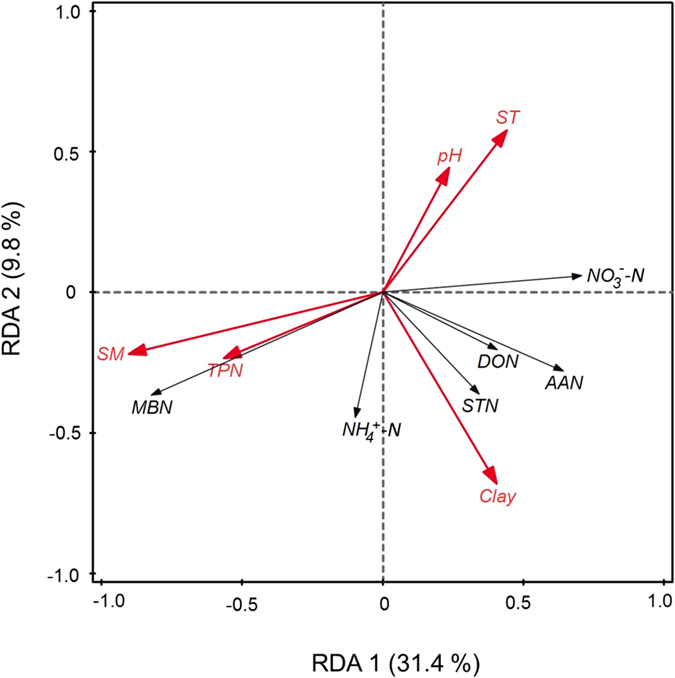
Biplot of redundancy analysis (RDA) of the relationships between six soil N stocks (black arrows) and other key variables (red arrows) in the topsoil under warming and altered precipitation regimes. Soil N pools: STN, soil total N; NO_3_^−^-N, nitrate-N; DON, dissolved organic N; AAN, amino acid N; MBN, microbial biomass N. Explanatory variables: TPN, total plant N content; SM, soil moisture; ST, soil temperature; Clay, soil clay content; pH, soil pH.

Redundancy analysis (RDA) was used to explore the relationship between the soil N stocks and environmental variables in the topsoil (Fig. 5). Axes 1 and 2 explained 31.4% and 9.8% of the total variation, respectively. According to the directions of arrows, NO_3_^−^-N had a highly negative correlation with SM (or TPN), while MBN had a positive correlation with SM (or TPN), and a highly negative correlation with ST (or pH). Among the five variables affecting soil N pools, the arrow for SM was the longest, showing that soil moisture accounted for the greatest proportion (26.3%) of the variance of soil N pools (Fig. 5; Table S3).

## Discussion

In our study, we observed significant changes in most soil N stocks in the topsoil, but no detectable changes in all measured soil N stocks in the subsoil and across the entire profile after 3 years of treatments. This suggested that soil N pools in the subsoil were not affected in the short period, which is partially inconsistent with our hypothesis (1). Thus, changes in soil N pools in the topsoil were further discussed.

Unexpectedly, we observed that warming significantly reduced soil MBN stock in the topsoil. This finding is inconsistent with two recent studies, which reported higher soil MBN stock with warming in the Tibetan grasslands^13^,^38^. In general, warming affects soil MBN by direct and indirect paths. On the one hand, when soil temperature increases close to the optimum temperature for growth under warming condition, the positive direct effects of warming enhance soil MBN stock by increased soil temperature^4^,^39^, especially in the alpine and arctic ecosystems. One the other hand, warming might indirectly aggravate water deficit through elevated evapotranspiration^19^,^28^,^40^, which inhibits microbial growth and leads to lower MBN stock. This is considered as a negative indirect effect of warming. In this region, the negative indirect effects are likely stronger than the positive direct effects, which can be proposed to explain why warming decreased soil MBN stock in this study. Firstly, compared with the large diurnal and seasonal temperature variations in this high-elevation ecosystems^20^, a rise in soil temperature of 1.6 °C in the study site is negligible to impact soil N pools. Secondly, especially under dry soil condition, soil moisture, rather than soil temperature, limits microbial growth. The observation that MBN stock in the topsoil was positively correlated with soil moisture supports this explanation (Fig. S1; Table S1). In addition, the difference in MBN between this study site and other Tibetan grasslands may be attributed to the different warming method as well. First, many studies have demonstrated that the peak daily temperature using Open Top Chambers (OTCs) was about 3–5 °C higher in the daytime, compared to the Infrared Lamps^26^,^41^. The increase in soil temperature using OTCs may be strong enough to enhance the MBN stock. Second, leaf litter accumulation and lack of wind could also lead to different results between OTCs and Infrared Lamps.

Our findings showed that warming increased NO_3_^−^-N stock in the topsoil, which supports results from past studies^19^,^21^. The higher soil NO_3_^−^-N stock may be caused by enhanced net nitrification by warming-induced drier condition (Fig. S3). In our study, we found that warming significantly decreased soil moisture by 2.3–7.3% at three depths. The drier soil under warmer condition has higher porosity, which is more likely to intensify air exchange and nitrification that converted NH_4_^+^-N and other forms of N to NO_3_^−^-N^42^. This explanation was supported by the decreased trends of NH_4_^+^-N, DON and AAN under warming (Fig. S3).

A reduction in NO_3_^−^-N, DON and AAN stocks in the topsoil under the increased precipitation treatment was detected. The finding is not consistent with previous studies, which have demonstrated that water addition enlarged soil N pools^43^,^44^. Likewise, some studies have shown that water amendment enhanced soil NO_3_^−^-N stock in grassland ecosystems^29^,^30^,^45^. There are four possible reasons why increased precipitation decreased dissolved N stocks in this study (Fig. S3). First, the mesic alpine grassland has a high soil water infiltration rate, compared with other grasslands, which was reported by Yang *et al*.^46^. The leaching rate could be aggravated to wash away the dissolved N pools in the wet plots^47^. Second, Rui *et al*.^19^ demonstrated that microbes prefer N immobilization over mineralization during low-temperature incubation of soils from a nearby alpine grassland. High moisture led to higher MBN in our study, which inferred that enhanced microbial biomass in the wet plots may consume more soil N by immobilization rather than release more soil N by mineralization. Third, mean soil moisture in the wet plots during the rainy days were up to 39% at 10 cm depth, approaching the denitrification condition reported^14^,^48^. Relatively high soil moisture is more likely to form an anaerobic environment to promote denitrification^48^. Higher denitrification rate in the wet plots may result in a drop in NO_3_^−^-N stock^49^. Fourth, many studies have demonstrated that the decrease in soil N pool was attributed to the enhanced plant uptake^31^,^50^. In our study, we did observe the increased N uptake in plants, which contributed to a reduction in the soil N concentrations (Fig. S2). This explanation was strongly supported by the negative correlation between total plant N uptake and NO_3_^−^-N, DON and AAN (Fig. 5).

Interestingly, AAN pool had a greater percentage change (averagely 22.5–28.5%) under altered precipitation regimes than other soil N pools in this study. This result is likely due to the comprehensive effects of plants and microbes. On one hand, plants absorb organic N, mainly in the form of AAN, in arctic and alpine regions^35^,^36^,^37^,^42^. On the other hand, microbes compete with plant roots to take up soil AAN^51^. Compared to other soil organic N pools, AAN is a preferred source for microbes to uptake because of its smaller molecular size^42^. Therefore, this finding implies that AAN pool may be very sensitive to climate change in this alpine grassland.

Principal components analysis and redundancy analysis illustrated that precipitation (or SM) dominated the response of soil N pools to climate change in the topsoil. This finding was in disagreement with our hypothesis (2) and a number of studies in the alpine regions which showed temperature as the limiting factor for soil N pools^27^,^52^. However, this finding is consistent with Baumann *et al*.^53^, who demonstrated soil moisture as a controlling factor for soil nitrogen and carbon contents across the Tibetan Plateau.

Why does precipitation, not temperature, modify soil N pools in the Tibetan alpine grassland? We proposed that plants, microbes and soil physical processes would be the main factors in determining soil N pools together, while these factors are controlled by precipitation (or SM) in Tibetan alpine grasslands. Firstly, Shi *et al*.^54^ showed that plant NPP in Tibetan alpine grasslands was primarily driven by precipitation, not temperature, in a transect experiment. The Tibetan alpine grasslands are mainly constrained by water availability^54^. When higher precipitation promotes plant NPP, roots uptake more nutrients from soil to support plant growth. Thus, soil N stocks were controlled by precipitation in this nitrogen-poor ecosystem. Likewise, Geng *et al*.^55^ reported that soil moisture best explains the large-scale patterns of soil respiration in Tibetan alpine grasslands. Soil moisture affects plant growth and microbial activity. On the one hand, enhanced plant growth needs more nutrients from soils. On the other hand, under higher precipitation condition, microbes compete with plants in taking up soil N, and sequentially decrease soil N stocks. Third, precipitation could also lead to loss of soil N by leaching or enhance soil N stocks by altering soil pH^14^,^46^. Thus, precipitation imposes greater influence on the soil nitrogen pools than warming in the mesic alpine grassland ecosystem.

Although the extent of warming and altered precipitation that we set exceeded the actual changes of temperature and precipitation in the Tibetan grassland, which may lead to uncertainties into our conclusions, the current conclusions are still highly credible. This is because the variation between the designed precipitation and actual precipitation is much smaller than the variation of temperature, which suggests that precipitation is more likely to change close to the treatment levels and potentially show significant impacts on soil N pools. What’s more, effects of warming on soil N pools were little in our study, even if the temperature rise was conducted much higher than the real change. Therefore, the uncertainties caused by the extent of change in temperature and precipitation should be weak.

## Methods

### Study site

The experiment was conducted at the Haibei National Field Observatory and Research Station for Alpine Grassland Ecosystem (Haibei Station). The station is located in the northeast of the Tibetan Plateau (101°19′E, 37°36′N, 3215 m a.s.l.). The site has a typical Plateau continental climate, which is affected by the southeast monsoon in summer and the Siberian cold in winter. From 1983 to 2013, mean annual air temperature is −1.1 °C, mean annual precipitation is 485 mm and over 84% of the precipitation is allocated to the growing season from May to September^56^. The soil developed is classified as Mat-Gryic Cambisols (Chinese Soil Taxonomy), and as *borolls* (USDA Soil Taxonomy). Soil total carbon and organic carbon concentrations at 0–10 cm depth are 78.2 g kg^−1^ and 63.1 g kg^−1^, respectively. Soil clay content, bulk density and pH at 0–10 cm depth are 65.3%, 0.8 g cm^−3^ and 7.8, respectively. The plant community is dominated by *Kobresia humilis, Festuca ovina, Elymus nutans, Poa pratensis, Carexscab rirostris, Scripus distigmaticus* and *Gentiana straminea*. Aboveground net primary production ranged from 237 to 503 g m^−2^ yr^−1^ from 1983 to 2012, according to the monitoring of our research group.

### Experimental design

Our experiment simulated the actual changes of temperature and precipitation in this Tibetan alpine grassland. The field experiment is a complete factorial design with temperature [control (C) and heated (H)] and precipitation [ambient (A), dry (D) and wet (W)] as treatment factors randomized in six blocks, resulting in 36 plots (two temperature levels × three precipitation levels × six replicates). Each plot is 2.2 m × 1.8 m with 4 m buffer zone between the plots.

The infrared heating system was set up in July 2011. Two medium-wave infrared heaters (1200 W, 220 V, 1 m long, 0.22 m wide) were fixed 1.6 m high above ground by stainless steel modules in each plot. Many studies have reported that the Tibetan alpine grassland has experienced a dramatic rise in air temperature by 0.20–0.51 °C per decade during the past years^10^,^11^,^12^. Thus, we designed to increase the soil surface temperature by approximately 2.0 °C per year, which is relatively higher than the actual trend of temperature in this region. Changes in precipitation were achieved by four “V” shaped transparent panlite sheet channels (PC-1151, Teijin chemical, Japan) in the altered precipitation plots, which were installed at an angle of 15°. According to the relationships among the minimum (353 mm), maximum (610 mm) and mean annual precipitation (488 mm) from the past 33 years of precipitation monitoring (from 1981–2014) in this site, we designed to increase and decrease 50% of annual precipitation as treatment levels. Water for higher precipitation plots (W) was drained from the lower precipitation plots (D), which was 50% of the ambient precipitation. To remove the possible effects of installations, dummy ones were installed for control plots. The soil around each plot had been trenched to 50 cm and lined with stainless steel sheets to prevent the movement of water and nutrients between plots.

### Soil sampling and measurements

Soil samples were taken using a drill at depths of 0–10 cm, 10–20 cm, 20–30 cm, 30–40 cm and 40–70 cm from four blocks on August 25, 2013. Three soil cores were randomly collected in each plot and were bulked as a composite sample and then passed through a 2-mm sieve and stored at 4 °C.

Soil NH_4_^+^-N, NO_3_^−^-N and DTN (including AAN) in the subsamples were extracted with 2 M KCl solutions (1:5 v/v soil/solution ratio) within two weeks in the laboratory^57^,^58^. Soil MBN was extracted with 0.5 M K_2_SO_4_ solutions (1:5 v/v soil/solution ratio) from the fumigated (24 hours) and non-fumigated soils using chloroform fumigation-extraction method with the conversion factor 0.45^59^. All Soil N extracts were measured with a Continuous Flowing Automatic Analyzer (CFAA) (Seal AutoAnalyzer 3 (AA3), Germany). Soil NH_4_^+^-N in the extract was determined by the method of the indophenol blue colorimetric analysis on the CFAA, and soil NO_3_^−^-N in the extract was measured after cadmium reduction from NO_2_^−^-N to NO_2_^−^-N, followed by sulfanilamide NAD reaction on the CFAA^14^. MBN (fumigated and unfumigated) and DTN in the extracts were determined by the potassium persulfate oxidation method on the CFAA^4^,^57^. DON was calculated as the difference between the DTN and the inorganic N (NH_4_^+^-N and NO_3_^−^-N). Soil AAN in the extract was determined by the fluorometric OPAME procedure^58^. Soil total N was determined on an Elemental Analyzer (Perkin Elmer instruments series II, USA).

Soil pH was measured in 1:5 (v/v) soil/water extracts using a pH Meter (FiveEasy FE20, Switzerland). Soil particle size was measured on a Laser Particle Size Analyzer (Mastersizer 2000, England). Soil temperature and moisture were monitored hourly using EM-50 devices and 5-TM sensors (Decagon devices, USA) at 5 cm, 10 cm and 20 cm depths and recorded by dataloggers.

### Data processing

Each soil N stock was calculated by each soil N pool concentration and bulk density at each depth (0–20 cm, 40–70 cm or 0–70 cm) using equation (1).


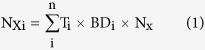


Where N_xi_ was soil N_x_ stock at depth i (kg m^−2^ or g m^−2^); T_i_ was soil layer thickness (cm), BD_i_ was bulk density (g cm^−3^); N_x_ was the concentration of each soil N pool at depth i (g kg^−1^ or mg kg^−1^).

### Statistical analysis

The main and interactive effects of warming and altered precipitation on soil temperature and moisture were analyzed by a repeated measure ANOVA. We used warming and precipitation treatments as fixed factors and sample date/block as random factors. The main and interactive effects of warming and altered precipitation on soil N stocks at each depth were analyzed using Linear Mixed Model (LMM, R package: nlme). Warming and precipitation treatments were included as fixed effects, and block was included as a random effect. Tukey’s HSD was used to determine if there were significant differences (α = 0.05) between individual treatments. Principal component analysis was determined to analyze the relationships among soil N pools across the treatments in the topsoil. Redundancy analysis was used to examine the relationship between soil N pools and environmental variables (soil moisture, soil temperature, soil pH, soil clay content and total plant N uptake) across the treatments in the topsoil. All statistical analyses were performed in R v. 3.1.1^60^.

## Additional Information

**How to cite this article**: Lin, L. *et al*. Precipitation overrides warming in mediating soil nitrogen pools in an alpine grassland ecosystem on the Tibetan Plateau. *Sci. Rep.* **6**, 31438; doi: 10.1038/srep31438 (2016).

## Supplementary Material

Supplementary Information

## Figures and Tables

**Table 1:** Summary of analysis of Linear Mixed Model (LMM) of the effects of warming (T), altered precipitation (P) and their interactions on soil nitrogen (N) stocks in the topsoil (0–20 cm), the subsoil (40–70 cm) and the entire soil profile (0–70 cm) (Overall).

Variables	Factors d.f.	Topsoil (0–20 cm)				Subsoil (40–70 cm)				Overall (0–70 cm)			
		B	T	P	T × P	B	T	P	T × P	B	T	P	T × P
		3	1	2	2	3	1	2	2	3	1	2	2
STN	F	1.757	0.325	0.358	0.934	1.070	0.076	0.946	3.043	1.804	0.102	0.408	1.723
	P	0.199	0.577	0.705	0.415	0.391	0.787	0.410	0.078	0.190	0.754	0.672	0.212
NH4^+^-N	F	4.760	2.547	1.620	0.159	1.147	0.245	0.642	0.156	4.426	1.995	1.373	0.353
	P	0.016*	0.131	0.231	0.854	0.362	0.628	0.540	0.857	0.020**	0.178	0.283	0.708
NO3^−^-N	F	2.849	10.976	25.552	0.614	0.606	0.001	0.201	0.431	2.020	3.306	5.739	0.643
	P	0.073	0.005**	0.000**	0.554	0.621	0.974	0.820	0.658	0.154	0.089	0.014*	0.540
DON	F	1.358	0.461	7.136	0.200	14.176	2.573	2.308	0.411	11.553	2.698	1.888	0.727
	P	0.293	0.508	0.007**	0.821	0.000**	0.130	0.134	0.670	0.000**	0.121	0.186	0.499
AAN	F	9.451	1.905	13.846	0.185	3.435	0.227	0.364	0.929	10.000	1.028	7.583	0.113
	P	0.001**	0.188	0.000**	0.833	0.044*	0.641	0.701	0.416	0.001**	0.327	0.005**	0.894
MBN	F	3.034	10.147	11.070	0.488	1.991	0.055	2.794	3.538	5.104	3.768	1.074	0.874
	P	0.062	0.006**	0.001**	0.623	0.159	0.817	0.093	0.055	0.012*	0.071	0.367	0.438

Warming and precipitation treatments were included as fixed effects, and block (B) was included as a random effect. STN, soil total nitrogen; SIN, soil inorganic N; NH_4_^+^-N, ammonium-N; NO_3_^−^-N, nitrate-N; SON, soil organic N; DON, dissolved organic N (2 M KCl extracts); AAN, amino acid N; MBN, microbial biomass N; d.f., degrees of freedom. ^**^P < 0.01; ^*^P < 0.05. Red asterisks indicate the positive effects; Blue asterisks indicate the negative effects.

## References

[b1] Norby R. J., Warren J. M., Iversen C. M., Medlyn B. E. & McMurtrie R. E. CO_2_ enhancement of forest productivity constrained by limited nitrogen availability. Proc. Natl. Acad. Sci. USA 107, 19368–19373 (2010). 20974944 10.1073/pnas.1006463107 PMC2984154

[b2] Zhang W. . Soil microbial responses to experimental warming and clipping in a tallgrass prairie. Global Change Biol. 11, 266–277 (2005).

[b3] Rustad L. E. . A meta-analysis of the response of soil respiration, net nitrogen mineralization, and aboveground plant growth to experimental ecosystem warming. Oecologia 126, 543–562 (2001). 10.1007/s004420000544 28547240

[b4] Belay-Tedla A., Zhou X. H., Su B., Wan S. Q. & Luo Y. Q. Labile, recalcitrant, and microbial carbon and nitrogen pools of a tallgrass prairie soil in the US Great Plains subjected to experimental warming and clipping. Soil Biol. Biochem. 41, 110–116 (2009).

[b5] Xu Z. H. & Chen C. R. Fingerprinting global climate change and forest management within rhizosphere carbon and nutrient cycling processes. Environ. Sci. Pollut. R. 13, 293–298 (2006). 10.1065/espr2006.08.340 17067023

[b6] Tian H. Q. . Patterns of soil nitrogen storage in China. Global Biogeochem. Cycles 20, GB1001 (2006).

[b7] Yang Y. H., Ma W. H., Mohammat A. & Fang J. Y. Storage, patterns and controls of soil nitrogen in China. Pedosphere 17, 776–785 (2007).

[b8] He N. P., Yu Q., Wu L., Wang Y. S. & Han X. G. Carbon and nitrogen store and storage potential as affected by land-use in a Leymus chinensis grassland of northern China. Soil Biol. Biochem. 40, 2952–2959 (2008).

[b9] Shi Y. . Organic and inorganic carbon in the topsoil of the Mongolian and Tibetan grasslands: pattern, control and implications. Biogeosciences 9, 2287–2299 (2012).

[b10] Xu Z. X., Gong T. L. & Li J. Y. Decadal trend of climate in the Tibetan Plateau-regional temperature and precipitation. Hydrol. Process. 22, 3056–3065 (2008).

[b11] Li L., Yang S., Wang Z. Y., Zhu X. D. & Tang H. Y. Evidence of warming and wetting climate over the Qinghai-Tibet Plateau. Arctic Antarct. Alpine Res. 42, 449–457 (2010).

[b12] Chen H. . The impacts of climate change and human activities on biogeochemical cycles on the Qinghai-Tibetan Plateau. Global Change Biol. 19, 2940–2955 (2013). 10.1111/gcb.12277 23744573

[b13] Li N., Wang G. X., Yang Y., Gao Y. H. & Liu G. S. Plant production, and carbon and nitrogen source pools, are strongly intensified by experimental warming in alpine ecosystems in the Qinghai-Tibet Plateau. Soil Biol. Biochem. 43, 942–953 (2011).

[b14] Liu G. S., Jiang N. H., Zhang L. D. & Liu Z. L. Soil physical and chemical analysis and description of soil profiles. China Standard Methods Press, Beijing, China 24, 266 (1996).

[b15] Lauber C. L., Strickland M. S., Bradford M. A. & Fierer N. The influence of soil properties on the structure of bacterial and fungal communities across land-use types. Soil Biol. Biochem. 40, 2407–2415 (2008).

[b16] Zhou X. Q. . Soil extractable carbon and nitrogen, microbial biomass and microbial metabolic activity in response to warming and increased precipitation in a semiarid Inner Mongolian grassland. Geoderma 206, 24–31 (2013).

[b17] Song B. . Light and heavy fractions of soil organic matter in response to climate warming and increased precipitation in a temperate steppe. PloS one 7, e33217 (2012). 22479373 10.1371/journal.pone.0033217 PMC3316559

[b18] Shen R. C., Xu M., Chi Y. G., Yu S. & Wan S. Q. Soil microbial responses to experimental warming and nitrogen addition in a temperate steppe of northern China. Pedosphere 24, 427–436 (2014).

[b19] Rui Y. C. . Warming and grazing affect soil labile carbon and nitrogen pools differently in an alpine meadow of the Qingha -Tibet Plateau in China. J. Soils Sediments 11, 903–914 (2011).

[b20] Yu C. Q., Shen Z. X. & Zhang X. Z., Sun, W. & Fu, G. Response of soil C and N, dissolved organic C and N, and inorganic N to short-term experimental warming in an alpine meadow on the Tibetan Plateau. The Scientific World Journal 2014, 152576 (2014). 24977179 10.1155/2014/152576 PMC4055494

[b21] Wang X. X. . Effects of short-term and long-term warming on soil nutrients, microbial biomass and enzyme activities in an alpine meadow on the Qinghai-Tibet Plateau of China. Soil Biol. Biochem. 76, 140–142 (2014).

[b22] Li N., Wang G. X., Gao Y. H. & Wang J. F. Warming effects on plant growth, soil nutrients, microbial biomass and soil enzymes activities of two alpine meadows in Tibetan Plateau. Pol. J. Ecol. 59, 25–35 (2011).

[b23] Zhang N. Y., Guo R., Song P., Guo J. X. & Gao Y. Z. Effects of warming and nitrogen deposition on the coupling mechanism between soil nitrogen and phosphorus in Songnen Meadow Steppe, northeastern China. Soil Biol. Biochem. 65, 96–104 (2013).

[b24] Rinnan R., Michelsen A. & Jonasson S. Effects of litter addition and warming on soil carbon, nutrient pools and microbial communities in a subarctic heath ecosystem. Appl. Soil Ecol. 39, 271–281 (2008).

[b25] Weedon J. T. . Summer warming accelerates sub‐arctic peatland nitrogen cycling without changing enzyme pools or microbial community structure. Global Change Biol. 18, 138–150 (2012).

[b26] Bai E. . A meta-analysis of experimental warming effects on terrestrial nitrogen pools and dynamics. New Phytol. 199, 431–451, doi: 10.1111/nph.12252 (2013). 23550663

[b27] Shaw M. R. & Harte J. Response of nitrogen cycling to simulated climate change: differential responses along a subalpine ecotone. Global Change Biol. 7, 193–210 (2001).

[b28] Liu W. X., Zhang Z. & Wan S. Q. Predominant role of water in regulating soil and microbial respiration and their responses to climate change in a semiarid grassland. Global Change Biol. 15, 184–195 (2009).

[b29] Lü X. T. & Han X. G. Nutrient resorption responses to water and nitrogen amendment in semi-arid grassland of Inner Mongolia, China. Plant Soil 327, 481–491 (2009).

[b30] Lü F.-M. . Carbon and nitrogen storage in plant and soil as related to nitrogen and water amendment in a temperate steppe of northern China. Biol. Fert. Soils 47, 187–196 (2010).

[b31] Luo Y. Q. . Progressive nitrogen limitation of ecosystem responses to rising atmospheric carbon dioxide. Bioscience 54, 731–739 (2004).

[b32] Jongen M., Lecomte X., Unger S., Fangueiro D. & Pereira J. S. Precipitation variability does not affect soil respiration and nitrogen dynamics in the understorey of a Mediterranean oak woodland. Plant Soil 372, 235–251 (2013).

[b33] Lipson D. & Nasholm T. The unexpected versatility of plants: organic nitrogen use and availability in terrestrial ecosystems. Oecologia 128, 305–316 (2001). 24549899 10.1007/s004420100693

[b34] Miller A. E. & Bowman W. D. Alpine plants show species-level differences in the uptake of organic and inorganic nitrogen. Plant Soil 250, 283–292 (2003).

[b35] Kielland K. Amino acid absorption by arctic plants: implications for plant nutrition and nitrogen cycling. Ecology 75, 2373–2383 (1994).

[b36] Schimel J. P. & Chapin I. S. F. Tundra plant uptake of amino acid and NH_4_ ^+^ nitrogen *in situ*: plants complete well for amino acid N. Ecology 77, 2142–2147 (1996).

[b37] Xu X. L., Ouyang H., Kuzyakov Y., Richter A. & Wanek W. Significance of organic nitrogen acquisition for dominant plant species in an alpine meadow on the Tibet plateau, China. Plant Soil 285, 221–231 (2006).

[b38] Zhang B. . Responses of soil microbial communities to experimental warming in alpine grasslands on the qinghai-tibet plateau. PloS one 9, e103859 (2014). 25083904 10.1371/journal.pone.0103859 PMC4118913

[b39] Sheik C. S. . Effect of warming and drought on grassland microbial communities. ISME J. 5, 1692–1700 (2011). 21451582 10.1038/ismej.2011.32 PMC3176507

[b40] Fu G., Shen Z. X., Zhang X. Z. & Zhou Y. T. Response of soil microbial biomass to short-term experimental warming in alpine meadow on the Tibetan Plateau. Appl. Soil Ecol. 61, 158–160 (2012).

[b41] Johnson C. P., Pypker T. G., Hribljan J. A. & Chimner R. A. Open top chambers and infrared lamps: A comparison of heating efficacy and CO_2_/CH_4_ dynamics in a northern Michigan peatland. Ecosystems 16, 736–748 (2013).

[b42] Chapin F. S. III, Moilainen L. & Kielland K. Preferential use of organic acid N by a non-mycorrhizal arctic sedge. Nature 361, 150–153 (1993).

[b43] Zeglin L. H. . Altered precipitation regime affects the function and composition of soil microbial communities on multiple time scales. Ecology 94, 2334–2345 (2013). 24358718 10.1890/12-2018.1

[b44] Landesman W. J. & Dighton J. Response of soil microbial communities and the production of plant-available nitrogen to a two-year rainfall manipulation in the New Jersey Pinelands. Soil Biol. Biochem. 42, 1751–1758 (2010).

[b45] Harpole W. S., Potts D. L. & Suding K. N. Ecosystem responses to water and nitrogen amendment in a California grassland. Global Change Biol. 13, 2341–2348 (2007).

[b46] Yang Y. H. . Significant soil acidification across northern China’s grasslands during 1980s-2000s. Global Change Biol. 18, 2292–2300 (2012).

[b47] Gu S. . Characterizing evapotranspiration over a meadow ecosystem on the Qinghai-Tibetan Plateau. J. Geophys. Res. 113, 693–702 (2008).

[b48] Weier K. L., Doran J. W., Power J. F. & Walters D. T. Denitrification and the dinitrogen/nitrous oxide ratio as affected by soil water, available carbon, and nitrate. Soil Sci. Soc. Am. J. 57, 66–72 (1993).

[b49] De Klein C. A. M. & Van Logtestijn R. S. P. Denitrification in grassland soils in the Netherlands in relation to irrigation, N-application rate, soil water content and soil temperature. Soil Biol. Biochem. 28, 231–237 (1996).

[b50] Yang H. J. . Plant community responses to nitrogen addition and increased precipitation: the importance of water availability and species traits. Global Change Biol. 17, 2936–2944 (2011).

[b51] Owen A. G. & Jones D. L. Competition for amino acids betweem wheat roots and rhizosphere microorganisms and the role of amino acids in plant N acquisition. Soil Biol. Biochem. 33, 651–657 (2001).

[b52] Wang S. P. . Effects of warming and grazing on soil N availability, species composition, and ANPP in an alpine meadow. Ecology 93, 2365–2376 (2012). 23236908 10.1890/11-1408.1

[b53] Baumann F., He J.-S., Schmidt K., Kuhn P. & Scholten T. Pedogenesis, permafrost, and soil moisture as controlling factors for soil nitrogen and carbon contents across the Tibetan Plateau. Global Change Biol. 15, 3001–3017 (2009).

[b54] Shi Y. . Field-based observations of regional-scale, temporal variation in net primary production in Tibetan alpine grasslands. Biogeosciences 10, 16843–16878 (2013).

[b55] Geng Y. . Soil respiration in Tibetan alpine grasslands: belowground biomass and soil moisture, but not soil temperature, best explain the large-scale patterns. PloS one 7, e34968 (2012). 22509373 10.1371/journal.pone.0034968 PMC3324551

[b56] Wang Y. H. . Non-growing-season soil respiration is controlled by freezing and thawing processes in the summer monsoon-dominated Tibetan alpine grassland. Global Biogeochem. Cycles 28, 1081–1095 (2014).

[b57] Jones D. L. & Willett V. B. Experimental evaluation of methods to quantify dissolved organic nitrogen (DON) and dissolved organic carbon (DOC) in soil. Soil Biol. Biochem. 38, 991–999 (2006).

[b58] Jones D. L., Owen A. G. & Farrar J. F. Simple method to enable the high resolution determination of total free amino acids in soil solutions and soil extracts. Soil Biol. Biochem. 34, 1893–1902 (2002).

[b59] Vance E. D., Brookes P. C. & Jenkinson D. S. An extraction method for measuring soil microbial biomass C. Soil Biol. Biochem. 19, 703–707 (1987).

[b60] R. Core Team (2013). R: A language and environment for statistical computing, R Foundation for Statistical Computing, Vienna, Austria. URL http://www.R-project.org/ ″.

