# Supplementary material for: Precipitation overrides warming in mediating soil nitrogen pools in an alpine grassland ecosystem on the Tibetan Plateau
Source: Sci Rep. 2016 Aug 16;6:31438. doi: 10.1038/srep31438 (PMC4985624; doi:10.1038/srep31438)

Precipitation overrides warming in mediating soil nitrogen pools in an alpine grassland ecosystem on the Tibetan Plateau

Li Lin[1], Biao Zhu[1], Chengrong Chen[2], Zhenhua Zhang[3], Qi-Bing Wang[4*], Jin-Sheng He[1,3*]

[1] *Department of Ecology, College of Urban and Environmental Sciences, and Key Laboratory for Earth Surface Processes of the Ministry of Education, Peking University, 100871, Beijing, China*

[2] *Environmental Futures Research Institute, Griffith School of Environment, Griffith University, 4111, Nathan, Queensland, Australia*

[3] *Key Laboratory of Adaptation and Evolution of Plateau Biota, Northwest Institute of Plateau Biology, Chinese Academy of Sciences, 23 Xinning Rd., 810008 Xining, China*

[4] *State Key Laboratory of Vegetation and Environmental Change, Institute of Botany, Chinese Academy of Sciences, 100093, Beijing, China*

Supplementary information

Table S1. Soil N stocks under warming, altered precipitation and their interactions in the topsoil (0-20 cm), the subsoil (40-70 cm) and the soil profile (Overall, 0-70 cm). C, control; H, heated; D, dry; HD, heated and dry; W, wet; HW, heated and wet. Different letters mean significant differences between factors at *P*<0.05 for each soil depth. Mean ±SE is shown in the table. STN, soil total nitrogen; NH_4^+-N, ammonium-N; NO_3^--N, nitrate-N; DON, dissolved organic N; AAN, amino acid N; MBN, microbial biomass N; d.f., degrees of freedom

N pools	Depth	Stocks					
		HD	D	H	C	HW	W
STN	Topsoil	0.80±0.04	0.87±0.07	0.78±0.10	0.84±0.03	0.82±0.02	0.76±0.03
(kg m^{-2})	Subsoil	0.31±0.03	0.44±0.03	0.47±0.08	0.36±0.05	0.37±0.02	0.32±0.07
	Overall	1.55±0.05	1.82±0.10	1.71±0.24	1.66±0.08	1.66±0.04	1.53±0.10
NH_4^+ -N	Topsoil	4.15±0.57	4.43±0.61	3.49±0.41	4.21±0.78	3.19±0.53	3.86±0.18
(g m^{-2})	Subsoil	2.93±0.31	2.86±0.50	2.26±0.49	2.44±0.54	2.28±0.53	2.75±0.56
	Overall	9.69±0.37	9.92±0.37	8.00±1.01	9.15±1.81	7.62±1.30	9.33±0.58
NO_3^--N	Topsoil	1.76±0.06 a	1.64±0.04 ab	1.56±0.06 b	1.48±0.06 b	1.45±0.03 b	1.27±0.06 c
(g m^{-2})	Subsoil	0.72±0.07	0.80±0.14	0.77±0.04	0.79±0.12	0.77±0.09	0.68±0.03
	Overall	3.47±0.19 a	3.30±0.19 a	3.24±0.13 a	3.16±0.14 ab	3.10±0.13 ab	2.71±0.14 b
DON	Topsoil	5.40±0.42 a	5.71±0.33 ab	4.77±0.20 ab	4.96±0.20 b	4.53±0.28 b	4.49±0.25 b
(g m^{-2})	Subsoil	3.03±0.46	3.19±0.53	2.91±0.43	3.44±0.32	3.46±0.37	3.67±0.27
	Overall	11.19±0.71	11.96±1.03	10.35±0.75	11.22±0.51	11.15±0.36	11.13±0.40
AAN	Topsoil	0.52±0.08 a	0.59±0.07 a	0.41±0.05 ab	0.46±0.07 ab	0.32±0.04 ab	0.34±0.06 b
(g m^{-2})	Subsoil	0.20±0.05	0.14±0.03	0.13±0.04	0.16±0.02	0.15±0.04	0.14±0.03
	Overall	0.92±0.13	0.97±0.11	0.74±0.11	0.85±0.11	0.64±0.12	0.67±0.13
MBN	Topsoil	6.41±0.30 c	8.03±0.66 bc	7.88±0.64 b	8.63±0.37 ab	8.76±0.43 ab	9.85±0.55 a
(g m^{-2})	Subsoil	2.80±0.33	3.25±0.14	2.87±0.37	2.13±0.32	2.29±0.09	2.72±0.28
	Overall	12.99±0.44	15.53±0.92	13.93±1.37	14.27±1.06	14.68±0.41	15.80±1.71

Table S2. Correlation coefficients (R^2 and *P*) calculated between pairs of factor scores and soil N stocks in the topsoil. $^{**}P < 0.01$; $^{*}P < 0.05$; $^{\dagger}P < 0.1$

**Soil N pools**	**PCA 1**		**PCA 2**	
	**R^2**	***P***	**R^2**	***P***
Soil total N (kg m^{-2})	0.157	**0.055****	0.290	**0.007****
NH_4^+-N (g m^{-2})	0.000	0.979	0.725	0.000
NO_3^--N (g m^{-2})	0.710	**0.000****	0.062	0.243
Dissolved organic N (g m^{-2})	0.574	**0.000****	0.117	0.102
Amino acid N (g m^{-2})	0.726	**0.000****	0.023	0.480
Microbial biomass N (g m^{-2})	0.513	**0.000****	0.028	0.431

Table S3. Summary of redundancy analysis (RDA) of the relationships between altered soil N stocks and other key variables in the topsoil under warming and altered precipitation regimes. $^{**}P < 0.01$; $^{*}P < 0.05$; $^{\dagger}P < 0.1$

**Key variables**	**Explains (%)**	**Pseudo-F**	***P***
Soil moisture (v/v %)	26.3	7.8	0.002^{**}
Total plant N content (g m^{-2})	12.7	3.2	0.016^{*}
Soil temperature (^{o}C)	10.4	2.6	0.032^{*}
Soil clay content (%)	10.4	2.5	$0.054^{\dagger}$
Soil pH	4.7	1.1	0.296

Fig S1. Effects of warming, altered precipitation and their interaction on NO_3^--N **(a)**, dissolved organic N (2 M KCl extracts) **(b)**, amino acid N **(c)** and microbial biomass N **(d)** stocks in the topsoil, the subsoil and the entire soil profile. A, ambient; D, dry; W, wet. Different letters mean significant differences between factors at P<0.05 for each soil depth. Mean ± SE is shown in the figure

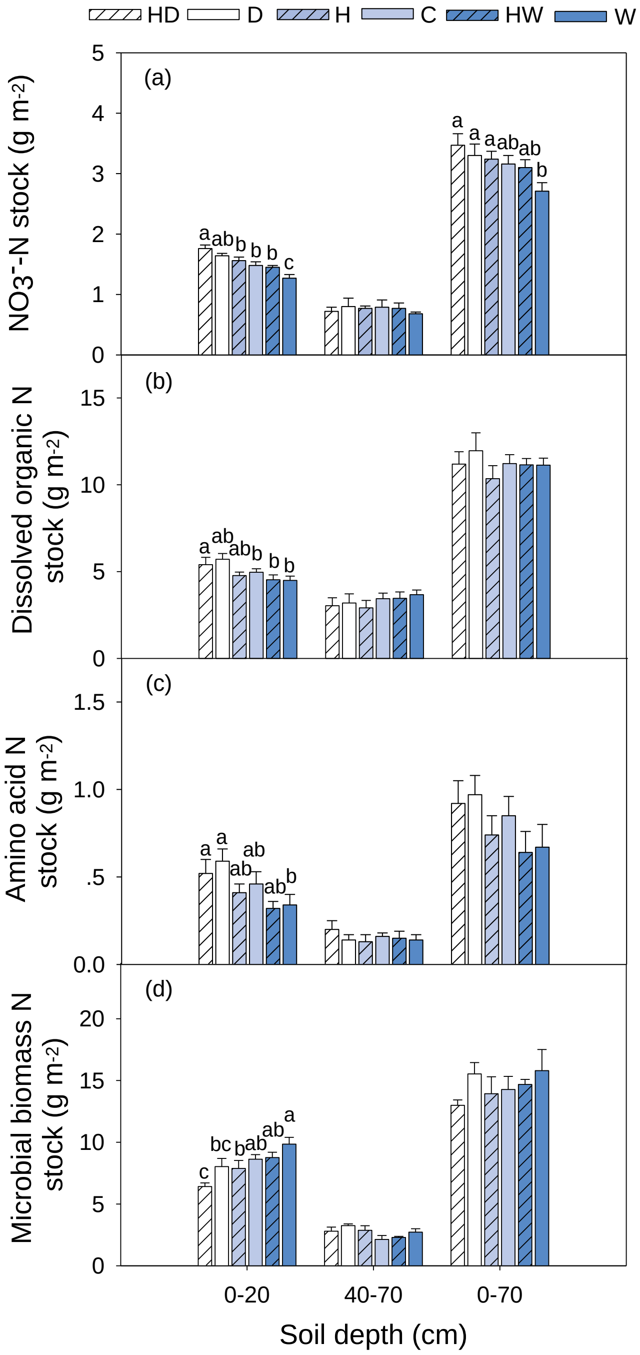

Fig S2. Effects of warming and altered precipitation on total plant N uptake. A, ambient; D, dry; W, wet. Different letters mean significant differences between factors at P<0.05

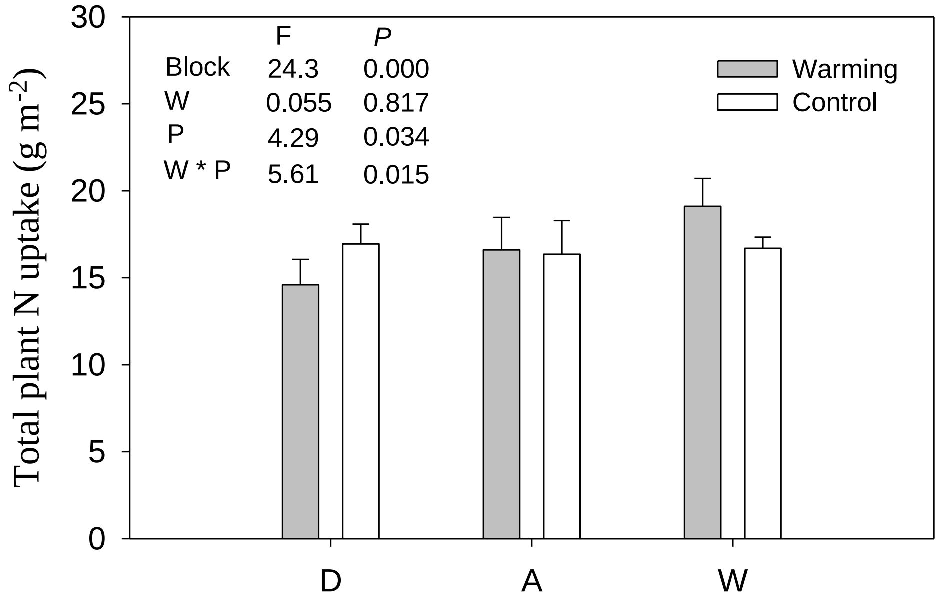

Fig S3. Schematic diagram showing how warming (a) and altered precipitation (b) affect soil N pools. ST, soil temperature; SM, soil moisture; NH_4^+, ammonium-N; NO_3^-, nitrate-N; DON, dissolved organic N (2 M KCl extracts); AAN, amino acid N; MBN, microbial biomass N; NPP, net primary production. Solid arrows, significant changes; Dashed arrows, insignificant trends.

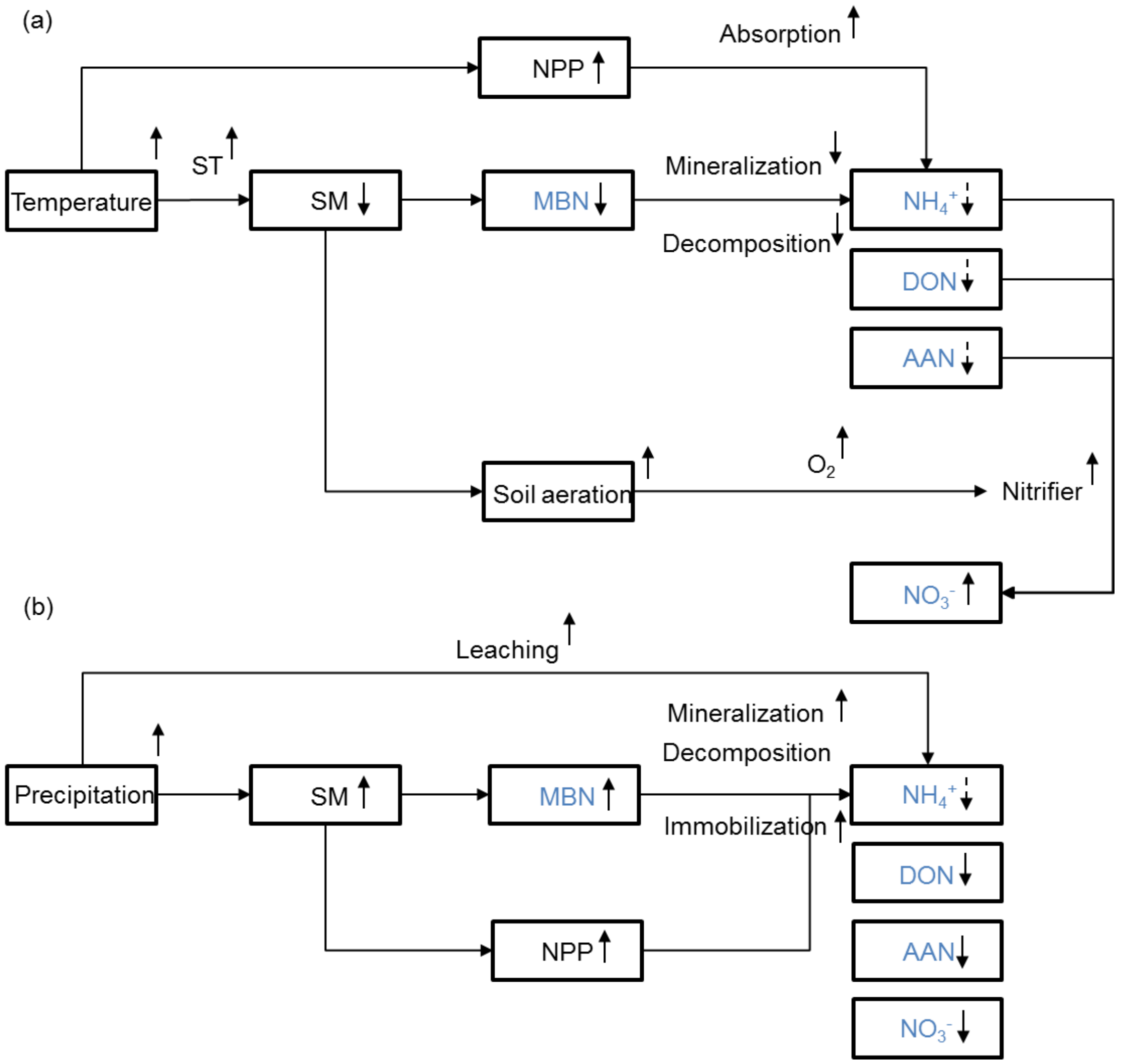

Supplement: Supplementary Information [file srep31438-s1.pdf]
